# Supplementary material for: Clinico-Radiological Phenotype of UBTF c.628G>A Pathogenic Variant-Related Neurodegeneration in Childhood: A Case Report and Literature Review
Source: Brain Sci. 2022 Sep 17;12(9):1262. doi: 10.3390/brainsci12091262 (PMC9496937; doi:10.3390/brainsci12091262)
Supplement: Supplementary file 1 [file brainsci-12-01262-s001.zip › Table S1.pdf]

Table S1. Clinical features of 15 individuals with *UBTF* c.628 G>A (p.Glu210Lys) pathogenic variant

Patient [Reference]	1 [3]	2 [3]	3 [3]	4 [3]	5 [3]	6 [3]	7 [3]	8 [4]	9 [4]	10 [4]	11[4]	12 [5]	13 [6]	14 [7]	15 [Index]
Gender	Female	Female	Female	Female	Female	Male	Female	Male	Male	Female	Female	Male	Male	Female	Male
Ethnicity	USA	Canada	USA	France	Israel	Russia	USA	European	Ashkenazi Jewish	European	European	Prague	Sri Lanka	Japan	Taiwan
Age at publication, year	23	17	16	19	19	11	8	11.6	6.2	33	12	13	12	19	14.3
Clinical manifestation															
DD noted at ≤ 2 years	No	Yes	Yes	No	No	No	Yes	Yes	No	Yes	Yes	Yes	Yes	Yes	Yes
Age at onset of neuroregression, yr	2.5	5	7	3	3	4	3.5	2.5	2-2.5	3	3	2	5.8	6	4
Initial motor regression	Yes	Yes	Yes	Yes	Yes	Yes	Yes	Yes	Yes	Yes	No	Yes	Yes	Yes	No
Initial speech regression	Yes	No	Yes	No	No	Yes	No	Yes	No	Yes	Yes	No	Yes	Yes	Yes
Microcephaly	Borderline	Acquired	Acquired	Acquired	Acquired	Acquired	No	No	Acquired	NA	NA	NA	No	NA	Acquired
Age at onset of epilepsy, year	No	15	5	14	No	No	No	No	No	No	No	6	11	No	10
Neurologic examination															
Deep tendon reflexes	NA	NA	NA	NA	NA	NA	NA	Brisk	Brisk	Brisk	Brisk	Brisk	Brisk	Brisk	Brisk
Spasticity	NA	Yes	Yes	Yes	Yes	Yes	Yes	Yes	Yes	Yes	Yes	Yes	Yes	Yes	Yes
Dystonia	Yes	Yes	No	Yes	No	Yes	No	Yes	No	Yes	Yes	Yes	Yes	Yes	Yes
Chorea	NA	NA	NA	Yes	No	NA	NA	Yes	No	No	No	No	Yes	No	No
Parkinsonism	NA	NA	NA	NA	Yes	NA	NA	NA	NA	NA	NA	NA	NA	Yes	Yes
Ataxia	Yes	No	Yes	No	No	No	Yes	Yes	Yes	Yes	Yes	Yes	Yes	NA	Yes
EEG	NA	Abn	Abn	Abn	Normal	Abn	Normal	Normal	NA	NA	Abn	Abn	Abn	Abn	Abn
Brain MRI															
Supratentorial cerebral atrophy	Yes	Yes	Yes	Yes	Yes	Yes	Yes	Yes	Yes	Yes	Yes	Yes	Yes	Yes	Yes
Cerebellar atrophy	Yes	Yes	Yes	Yes	No	No	Yes	Yes	Yes	Yes	Yes	No	Yes	No	Yes
Diffuse WM T2 hyperintensity	Yes	Yes	Yes	Yes	Yes	Yes	Yes	Yes	NA	NA	NA	Yes	Yes	Yes	Yes
Thalamus involvement	NA	NA	NA	NA	NA	NA	NA	NA	NA	NA	NA	Yes	NA	NA	Yes
Clinical Outcome															
Profound intellectual disability	Yes	Yes	Yes	Yes	Yes	Yes	Yes	Yes	Yes	Yes	Yes	Yes	Yes	Yes	Yes
Non-verbal	Yes	Yes	Yes	Yes	Yes	Yes	Yes	No	Yes	Yes	Yes	Yes	Yes	NA	Yes
Non-ambulatory	Yes	Yes	Yes	Yes	Yes	Yes	No	Yes	No	Yes	Yes	Yes	Yes	NA	Yes

Abn: Abnormal; DD: Developmental delay; EEG: electroencephalography; MRI: magnetic resonance imaging; NA: not available; WM: white matter; yr: year
